# Patterns of recommended vaccine receipt among women ages 24–45 years: a cross-sectional analysis

**DOI:** 10.1186/s12889-021-11340-4

**Published:** 2021-07-01

**Authors:** Mallory K. Ellingson, Carlos R. Oliveira, Sangini S. Sheth, Erin L. Sullivan, Ashlynn Torres, Eugene D. Shapiro, Linda M. Niccolai

**Affiliations:** 1 Department of Epidemiology of Microbial Diseases, Yale School of Public Health, New Haven, CT USA; 2 Department of Pediatrics, Yale School of Medicine, New Haven, CT USA; 3 Department of Obstetrics, Gynecology & Reproductive Sciences, Yale School of Medicine, New Haven, CT USA

**Keywords:** Adult vaccination, Immunization, Influenza vaccine, HPV vaccine, Women’s health

## Abstract

**Background:**

Vaccine receipt among mid-adults remains low, with only one quarter of adults being up to date for all recommended vaccines. It is important to understand the myriad factors that influence vaccine receipt among mid-adult women to address these low rates.

**Methods:**

We conducted a cross-sectional analysis of data from women ages 24–45 years collected as part of an ongoing case-control study of the effectiveness of HPV vaccine. We examined associations between demographic characteristics and healthcare utilization and receipt of individual vaccines and combinations of multiple vaccines using logistic regression analyses for three routinely recommended vaccines: tetanus, influenza and HPV.

**Results:**

Among the 309 women enrolled in the study, only 19 (6.2%) were up to date for all three recommended vaccines and 41 (13.3%) had not received any of the recommended vaccines. A greater number of health care visits in the past year was associated with receipt of influenza (aOR = 6.37, 95% CI = 2.53, 16.1) and tetanus (aOR = 2.17, 95% CI = 1.14, 4.12) vaccines. White women were more likely to have received HPV vaccine (aOR = 2.39, 95% CI = 1.07, 5.36).

**Conclusions:**

Uptake of recommended vaccines is low among young and mid-adult women. There is a need for greater understanding of the underlying factors influencing vaccine receipt in this population.

## Introduction

Routine vaccinations are an important part of preventive health throughout the life course. However, vaccine receipt drops significantly after childhood despite the routine recommendation for immunization with several vaccines during adulthood [1]. In 2017, only 25% of adults over the age of 19 were up to date on all age-appropriate routinely recommended vaccines in the United States, not including the human papillomavirus vaccine (HPV), and fewer than 50% of women had received at least one dose of the HPV vaccine [2]. In the last influenza season, less than 50% of individuals over the age of 18 years received the seasonal influenza vaccine [3].

There are myriad potential explanations for low adult immunization rates. Individual attitudes about vaccines can contribute substantially and can vary by each vaccine. Another is the wide variation among recommendations for different adult vaccines. The influenza vaccine is recommended annually while a tetanus-toxoid containing vaccine [tetanus-diphtheria (Td) or tetanus, diphtheria and acellular pertussis (Tdap)] is recommended every 10 years and Tdap is recommended during each pregnancy [1]. HPV vaccine recommendations vary based on the age at initiation, and other vaccines are only recommended for specific risk groups (e.g., pneumococcal polysaccharide vaccine for individuals under 64 with chronic medical or immunocompromising conditions) [1]. Recently, the Advisory Committee on Immunization Practices (ACIP) has also recommended shared clinical decision-making for several vaccines, including the HPV vaccine for adults over the age of 26 [4], pneumococcal conjugate vaccine for adults over the age of 65 without other indications for the vaccine [5], and the meningococcal serogroup B vaccine [6]. However, what “shared clinical decision-making” means remains unclear to many physicians [6, 7]. In addition to varying types of recommendations (e.g. routine vs. targeted), adult immunizations are generally not mandatory at the national or state level in the United States. Lack of knowledge and confusion about recommendations among both patients and clinicians may further contribute to low adult immunization rates.

Also of concern is that racial and ethnic disparities in adult vaccine receipt have persisted, with white adults generally having higher immunization rates than racial and ethnic minorities [2, 8]. Reasons for these racial and ethnic disparities are still not entirely understood [8–10]. Decisions about whether to accept a vaccine do not occur in a vacuum. A number of factors including socioeconomic status, healthcare access and utilization, and opinions or knowledge about other recommended vaccines may influence an individual’s ability or decision to be vaccinated [11]. Currently all vaccines recommended by the ACIP are required to be covered by private insurance with no copay; however, for individuals with public insurance or no insurance coverage, the price and availability of adult vaccines can vary [12]. Therefore, it is important not to just assess receipt of each individual recommended vaccine, but combinations of multiple vaccines among adults and the factors that impact these patterns to disentangle these multiple influences.

Among a sample of mid-adult women (ages 24–45 years), we evaluated patterns of vaccine receipt for three routinely recommended vaccines in this age group: seasonal influenza vaccine, tetanus vaccine, and HPV vaccine. Additionally, we evaluated how these receipt patterns varied by demographic and healthcare utilization factors known or hypothesized to be related to vaccine receipt.

## Methods

Data included in this study were collected as part of an ongoing case-control study in New Haven County, Connecticut during April 2016 and December 2019. Briefly, the purpose of the parent case-control study is to evaluate the real-world effectiveness of HPV vaccines as used in clinical practice. Cases are women diagnosed with high-grade cervical lesions reported to a statewide registry per mandatory reporting requirements. This population-based registry includes all women who reside in the catchment area of New Haven County regardless of the location or type of medical practice or pathology laboratory. The women included in this analysis were control participants (women with normal cytology), matched to cases on age, date of test, and medical practice, as they were selected to be more representative of the broader population of mid-adult women in the study area than the cases. These women were identified from a hospital-based cytology database and matched to cases (women with biopsy-confirmed high-grade cervical lesions) on age, date of testing, and medical practice. Potential participants were sent an invitation letter and asked to provide written informed consent to participate. Following enrollment, women received a brief survey to collect selected demographic information (age, race/ethnicity, education, income, insurance status, and pregnancy history) and healthcare utilization information, including types of provider visited and number of healthcare visits in the past year. The participation rate was 68%. Medical-record verified vaccine history was collected for all participants. Additionally, women were asked to provide the name of all previous healthcare providers since adolescence. Trained research staff reviewed all medical records electronically or by visiting the medical facility for any reported providers and reviewing the paper charts, allowing us to obtain verified vaccine history from multiple providers for some participants. Additional data collected from electronic medical records included the average number of healthcare visits per year over the course of the woman’s medical record.

For the purposes of this analysis, we defined women as ‘vaccinated’ for each of the three routinely recommended vaccines as follows: influenza vaccine receipt in the same influenza season as survey administration or in the prior influenza season, at least one dose of a tetanus-containing vaccine in the 10 years prior to survey administration, and at least one dose of HPV vaccine at any time. Women were considered completely vaccinated if they were vaccinated with all three of the routinely recommended vaccines. We acknowledge that a subset of women in our study (women over the age of 35) were not eligible to receive the HPV vaccine at any point in their life prior to the 2019 change in the ACIP recommendation to include women over the age of 26. As these women did have an opportunity to be vaccinated within the study population, we did not alter our definition of vaccinated for women over the age of 36, but did conduct an analysis evaluating vaccine coverage by age.

All analysis was conducted using R software package (v. 3.1.6) with α = 0.05. Descriptive analyses were conducted for all sociodemographic, healthcare utilization, and vaccine receipt variables. *P*-values were calculated using chi-square tests to evaluate the association between vaccinated status both for each individual vaccine and for all recommended vaccines. Adjusted odds ratios (aOR) and 95% confidence intervals (95% CI) were calculated using multivariable logistic regression. The models included terms for all socio-demographic and healthcare utilization factors that were a priori determined to potentially influence vaccine receipt based on a review of the literature: race/ethnicity, age, education, income, pregnancy history, insurance status, number of healthcare visits in the past year and type of healthcare provider seen (PCP and OBGYN). Age was dichotomized based on whether or not women were eligible to receive the HPV vaccine prior to the June 2019 change in the ACIP recommendation to include women ages 26–45. Healthcare visits were defined as any reported visit with a healthcare provider in the past year (≥1) and no visits with a healthcare provider as to capture those women who had any interaction with the healthcare system versus those who did not. This study was approved by the Yale University Institutional Review Board.

## Results

At the time of this analysis, 309 controls were enrolled and were included in subsequent analyses. These women were predominantly white (50.5%), 24–35 years of age (75.4%), privately insured (70.5%), and had at least a college degree (58.9%) (Table 1). Nearly all women averaged at least one visit to a provider annually (92.9%). More than half of women reported having both an obstetrician/gynecologist (OB/GYN) and a primary care provider (PCP) (57.6%). Approximately 30% reported only having an OB/GYN (31.1%). Less than half of women reported a history of pregnancy (45.3%). We were able to verify vaccine history in more than one record for approximately three-quarters of women (73.1%).

**Table 1:** Characteristics of study participants

	All Participants (*N* = 309)
	N (%)
**Age (years)**	
24–35	223 (75.4)
36–45	76 (24.6)
**Race / Ethnicity**	
White	156 (50.5)
Black	55 (17.8)
Hispanic	35 (11.3)
Other / Mixed	63 (20.4)
**Education**	
< College Graduate	124 (40.1)
≥ College Graduate	182 (58.9)
Missing/Not Provided	3 (1.0)
**Household Income**	
< $50,000	174 (56.3)
≥ $50,000	110 (35.6)
Missing/Not Provided	25 (8.1)
**Insurance Status**	
Private insurance	215 (70.5)
Public or military insurance	71 (23.3)
Uninsured	10 (3.3)
Missing/Not Provided	9 (3.0)
**History of Pregnancy**	
Yes	140 (45.3)
No	165 (53.4)
Missing/Not Provided	4 (1.3)
**Number of visits in past year (self-reported)**	
1	58 (19.1)
2	49 (16.2)
3	34 (11.2)
4+	56 (18.5)
Don’t know	106 (35.0)
**Average annual provider visits (electronic health record)**	
< 1	15 (4.9)
≥ 1	287 (92.9)
Missing	7 (2.2)
**Type of healthcare providers visited (self-reported)**	
OBGYN & PCP	178 (57.6)
OBGYN only	96 (31.1)
PCP only	11 (3.6)
Neither	24 (7.8)

Approximately one-third of women received an influenza vaccine in the most recent season or at least one dose of the HPV vaccine, however, nearly 70% of women had received a tetanus-containing vaccine in the previous 10 years (Table 2). Women between 36 and 45 had higher levels of being vaccinated with the influenza vaccine (24–35: 25.8% vs. 36–45: 44.7%, *p* = 0.003) and lower levels of being up to date for the HPV vaccine (24–35: 45.5% vs. 36–45: 10.5%, *p* < 0.001) compared to women 24–35. African-American/Black women and Hispanic women also reported higher levels of being vaccinated with a tetanus-containing vaccine (85.5 and 80.0%, respectively) compared to white women (62.2%, *p*-value = 0.004). History of pregnancy was not significantly associated with vaccine receipt for any of the three vaccines evaluated. The proportion of women who were vaccinated with the influenza vaccine increased with the number of healthcare visits a woman reported, with more than half of women who reported at least three visits receiving the influenza vaccine (55.9%) compared to 10% of women who only reported one visit (*p* < 0.001). Few women were completely vaccinated (*n* = 19, 6.2%), and a small but substantial portion of women had received none of the recommended vaccines (*n* = 41, 13.3%; Table 3).

**Table 2:** Vaccine receipt by demographic characteristics

	Received Vaccine					
	Influenza		Tetanus		HPV	
	N (%)	*p*-value	N (%)	*p*-value	N (%)	*p*-value
**Total (*N*** **= 309)**	94 (30.4)		219 (70.9)		114 (36.9)	
**Age (years)**		0.003		0.29		< 0.001
24–35	60 (25.8)		161 (69.1)		106 (45.5)	
36–45	34 (44.7)		58 (76.3)		8 (10.5)	
**Race / Ethnicity**		0.19		0.004		0.37
White	46 (29.5)		97 (62.2)		64 (41.0)	
Black	21 (38.2)		47 (85.5)		19 (34.5)	
Hispanic	6 (17.1)		28 (80.0)		13 (37.1)	
Other / Mixed	21 (33.3)		47 (74.6)		18 (28.6)	
**Education**		0.72		0.05		0.98
< College	40 (32.3)		96 (77.4)		46 (37.1)	
≥ College Graduate	54 (29.7)		121 (66.5)		66 (36.3)	
Missing/Not Provided	0 (0.0)		2 (66.7)		2 (66.7)	
**Household Income**		0.10		0.97		0.39
< $50,000	49 (28.2)		125 (71.8)		69 (39.7)	
≥ $50,000	42 (38.2)		78 (70.9)		35 (31.8)	
Missing	3 (12.0)		16 (64.0)		10 (40.0)	
**Insurance Status**		0.98		0.09		0.76
Private insurance	66 (30.7)		148 (68.8)		81 (37.7)	
Public and military insurance	21 (29.6)		52 (73.2)		24 (33.8)	
Uninsured	3 (30.0)		10 (100.0)		3 (30.0)	
Missing	3 (33.3)		6 (66.7)		3 (33.3)	
**History of Pregnancy**		1.0		0.05		0.13
Yes	42 (30.0)		107 (76.4)		45 (14.8)	
No	50 (30.3)		108 (65.5)		68 (22.3)	
**Number of visits in past year (self-reported)**		< 0.001		0.44		0.33
1	6 (10.3)		36 (62.0)		19 (32.8)	
2	17 (34.7)		36 (73.5)		16 (32.7)	
3	19 (55.9)		27 (79.4)		14 (41.2)	
4+	19 (33.9)		41 (73.2)		17 (30.4)	
Don’t know	33 (31.1)		76 (71.7)		47 (44.3)	
**Average annual provider visits (electronic health record)**		0.21		0.84		0.10
< 1	2 (13.3)		10 (66.7)		9 (60.0)	
≥ 1	92 (32.1)		208 (72.5)		102 (35.5)	
Missing	0 (0.0)		1 (14.3)		3 (42.9)	
**Type of healthcare providers visited (self-reported)**		0.31		0.46		0.70
OBGYN & PCP	61 (34.3)		127 (71.4)		70 (39.3)	
OBGYN only	26 (27.1)		71 (74.0)		32 (33.3)	
PCP only	2 (18.2)		7 (63.6)		2 (27.3)	
Neither	5 (20.8)		14 (58.3)		9 (37.5)

**Table 3:** Number of vaccines receivedA by demographic characteristics

	All Three	Any Two	Any One	None	
	N (%)	N (%)	N (%)	N (%)	***p***-value
**Total (*N*** **= 309)**	19 (6.2)	121 (39.2)	128 (41.4)	41 (13.3)	
**Age (years)**					0.46
24–35	13 (5.6)	96 (41.2)	96 (41.2)	28 (12.0)	
36–45	6 (7.9)	25 (32.9)	32 (42.1)	13 (17.1)	
**Race / Ethnicity**					0.23
White	12 (7.7)	51 (32.7)	69 (44.2)	24 (15.4)	
Black	3 (5.5)	28 (50.9)	22 (40.0)	2 (3.6)	
Hispanic	2 (5.7)	13 (37.1)	15 (42.9)	5 (14.3)	
Other / Mixed	2 (3.2)	29 (46.0)	22 (34.9)	10 (15.9)	
**Education**					0.37
No College	8 (6.5)	55 (44.4)	48 (38.7)	13 (10.5)	
College Graduate	11 (6.0)	64 (35.2)	80 (44.0)	27 (14.8)	
Missing/Not Provided	0 (0.0)	2 (66.7)	0 (0.0)	1 (33.3)	
**Household Income**					0.58
< $50,000	10 (5.7)	73 (42.0)	67 (38.5)	24 (13.8)	
≥ $50,000	9 (8.2)	40 (36.4)	48 (43.6)	13 (11.8)	
Missing/Not Provided	0 (0.0)	8 (32.0)	13 (52.0)	4 (16.0)	
**Insurance Status**					0.74
Private insurance	14 (6.5)	83 (38.6)	87 (40.4)	13 (14.4)	
Public or military insurance	4 (5.6)	27 (38.0)	31 (43.7)	9 (12.7)	
Uninsured	0 (0.0)	6 (60.0)	4 (40.0)	0 (0.0)	
Missing/Not Provided	0 (0.0)	3 (33.3)	6 (66.7)	0 (0.0)	
**History of Pregnancy**					0.22
Yes	7 (7.3)	61 (34.5)	51 (46.1)	21 (12.2)	
No	12 (5.0)	57 (43.6)	76 (36.4)	20 (15.0)	
**Number of visits in past year (self-reported)**					0.03
1	0 (0.0)	14 (24.1)	33 (56.9)	11 (19.0)	
2	4 (8.2)	19 (38.8)	19 (38.8)	7 (14.3)	
3	5 (14.7)	18 (52.9)	9 (26.5)	2 (5.9)	
4+	3 (5.4)	23 (41.1)	22 (39.3)	8 (14.3)	
Don’t know	7 (6.6)	46 (43.4)	43 (40.6)	10 (9.4)	
**Type of healthcare providers visited (self-reported)**					0.41
OBGYN & PCP	12 (6.7)	76 (42.7)	70 (39.3)	20 (11.2)	
OBGYN only	4 (4.2)	37 (38.5)	43 (44.8)	12 (12.5)	
PCP only	3 (9.1)	5 (18.2)	2 (45.5)	1 (27.3)	
Neither	2 (8.3)	6 (25.0)	10 (41.7)	6 (25.0)	
**Average annual provider visits (electronic health record)**					0.49
< 1	2 (13.3)	5 (33.3)	5 (33.3)	3 (20.0)	
≥ 1	17 (5.9)	115 (40.1)	121 (42.1)	34 (11.8)	
Missing	0 (0.0)	1 (14.3)	2 (28.6)	4 (57.1)	

When controlling for the other socio-demographic and healthcare utilization factors in the model, older age (35–45 years of age) and more than one annual visit to a doctor were positively and statistically significantly associated with being up to date for influenza vaccine (age: aOR = 2.59, 95% CI = 1.35, 4.97; visits: aOR = 6.37, 95% CI =2.53, 16.1) (Table 4). More than one visit and reporting an OB/GYN was positively associated with tetanus vaccine receipt (visits: aOR = 2.17, 95% CI = 1.14, 4.12; OB/GYN: aOR = 2.44, 95% CI = 1.01, 5.90). White women were more than twice as likely to receive the HPV vaccine as non-white women (aOR = 2.39, 95% CI = 1.07, 5.36), but older women (35–45 years old) were significantly less likely to receive the HPV vaccine compared to their younger counterparts (aOR = 0.16, 95% CI = 0.07, 0.37).

**Table 4:** Adjusted odds of vaccine receipt^a^

	Received Influenza Vaccine	Received Tetanus Vaccine	Received HPV Vaccine
	aOR (95% CI)	aOR (95% CI)	aOR (95% CI)
**Race**^**b**^			
White	0.74 (0.35, 1.61)	0.56 (0.25 1.27)	**2.39 (1.07, 5.36)**
Black	1.22 (0.51, 2.96)	1.87 (0.68, 5.14)	1.69 (0.69, 4.12)
Hispanic	0.44 (0.14, 1.61)	2.09 (0.63, 6.09)	1.86 (0.67, 5.15)
**Age**			
24–35 years	REF	REF	REF
36–45 years	**2.59 (1.35, 4.97)**	1.65 (0.81, 3.33)	**0.16 (0.07, 0.37)**
**Education**			
No college degree	REF	REF	REF
At least a college degree	0.69 (0.33, 1.44)	0.88 (0.41, 1.90)	0.69 (0.34, 1.40)
**Income**			
< $50,000	0.62 (0.32, 1.22)	0.81 (0.42, 1.55)	1.18 (0.62, 2.22)
≥ $50,000	REF	REF	REF
**History of pregnancy**	0.83 (0.46, 1.50)	1.37 (0.74, 2.53)	0.74 (0.41, 1.32)
**Insurance**^**c**^			
Private Insurance	0.60 (0.17, 2.03)	0.28 (0.05, 1.46)	1.24 (0.36, 4.28)
Public Insurance	0.53 (0.15, 1.84)	0.23 (0.05, 1.22)	0.93 (0.27, 3.25)
**Number of visits in the past year**			
≥ 1	**6.37 (2.53, 16.1)**	**2.17 (1.14, 4.12)**	1.31 (0.68, 2.55)
< 1	REF	REF	REF
**Reported seeing an OBGYN**	1.96 (0.75, 5.12)	**2.44 (1.01, 5.90)**	1.11 (0.44, 2.76)
**Reported seeing a PCP**	1.42 (0.79, 2.58)	1.07 (0.59, 1.93)	1.53 (0.86, 2.72)

^a^ The statistical models included all covariates listed in the table.

^b^ Reference group is participants of any other race/ethnicity besides the listed race/ethnicity

^c^ Reference group is participants with any other type of insurance besides the listed insurance

## Discussion

In this analysis from an ongoing case-control study that collected medical record verified vaccine histories of 309 women**,** we found that less than 10 % of women in the study population were completely vaccinated for this age group. Vaccine receipt of the individual vaccines was slightly lower in our sample than the national average of 36% coverage for influenza and 42% coverage for HPV vaccines, with only about 30% of women having received either the influenza vaccine or the HPV vaccine, but similar for tetanus vaccines [2]. Additionally, we found significant differences in receipt by race, age and self-reported number of healthcare visits in the past year. These results provide useful insight into patterns of vaccination coverage among adult women and potential avenues to address these disparities.

Women ages 36–45 years were more likely to be vaccinated with the influenza vaccine, whereas they were less likely to be vaccinated with the HPV vaccine when controlling for other factors. It is not surprising that these women were less likely to have received the HPV vaccine, given that some women in this population would not have been age-eligible for vaccination prior to the recent expansion to include women 27–45 years old for shared clinical decision making [4]. Influenza vaccine coverage tends to be higher among older adults [2, 13], presumably due to increased interaction with the healthcare system or increased recommendation of the vaccine based on comorbidities. However, it is unclear from our data why vaccine receipt would be higher among adults aged 36–45 years in this population, even after controlling for recent interactions with the healthcare system. This suggests that there might be additional barriers to vaccine receipt among younger women beyond having less interaction with the healthcare system.

Racial differences in vaccine receipt were observed in this population as well. White women were more likely to have received the HPV vaccine, even in adjusted analyses when we controlled for potential socio-economic factors and healthcare utilization. The social and economic determinants of vaccine receipt are complex, and the racial and ethnic differences in receipt in this population may be due to additional factors that were not measured in this study. In one study of the role of race and influenza vaccine uptake, authors found that Black adults were less likely to have sought out the vaccine and among those who did receive a vaccine, were much less likely than their White counterparts to have received the vaccine in a doctor’s office, regardless of education or poverty [10]. Similar to our study, an analysis of National Health Interview Survey data found that disparities in adult vaccine receipt persisted even once you controlled for other socioeconomic factors such as education, health insurance and number of physician visits in the past year [8]. Another possible explanation is that provider recommendation patterns may differ by patients’ race/ethnicity, leading to varying receipt among different populations independent of socio-economic status or healthcare utilization, but this has not been adequately studied. Unfortunately, a more nuanced understanding of racial and socioeconomic disparities in vaccination is beyond the scope of this study that was not designed to address this topic specifically. Therefore, further targeted research is needed to understand the full breadth of factors that influence receipt among different racial and ethnic groups.

Administration of age appropriate immunizations are recommended as part of annual preventive healthcare visits for women [14]. In our study, women who reported more than one healthcare visit in the past year were more likely to have received influenza and tetanus vaccines. Additionally, women who reported an OBGYN were more likely have received the tetanus vaccine, which may be related to the recommendation to receive Tdap vaccination during each pregnancy. These findings suggest that each interaction with the healthcare system does present an additional opportunity for immunization, particularly if the interactions occurred during an influenza season. However, missed opportunities for immunization persist. A missed opportunity is any visit to a healthcare provider where a vaccine could have been provided but was not [15]. One study estimated that eliminating missed opportunities for adults could improve influenza vaccine coverage up to 23% nationally [16]. In our study, even among those who reported three visits in the past year, influenza vaccination coverage was only 56%. Thus, though there is no formal requirement by which providers must review and recommend vaccines at each health care visits, incorporating best practices to treat vaccination as a vital sign that is checked at each visit could help to reduce missed opportunities. There is a need to engage providers of all specialties, but particularly those who provide routine preventive care such as primary care physicians and OB/GYNs in vaccine promotion year-round so that every healthcare visit represents an opportunity to be vaccinated.

The primary limitation of this study is a potential lack of generalizability. According to a 2013 report, approximately 22% of women over the age of 18 do not see a healthcare provider at all in a given year [17], whereas all participants in our study had at least one healthcare visit prior to study enrollment. Therefore, we may be over-estimating vaccine coverage in this population compared to the general population. However, for the women included in this study we were able to obtain vaccination history through a robust medical record review. Although medical record review of immunization history is not a perfect measure – both influenza and tetanus vaccines can be administered in a pharmacy and may not be recorded in a medical record and therefore could be underestimated in our records – this is still a strong advantage over other similar studies that typically rely on self-reported vaccine history. Additionally, there was substantial missing data for our self-reported measure of healthcare visits in the past year and we were limited in our ability to verify this self-reported healthcare utilization variable in the medical records.

## Conclusions

This study highlights the need for a greater focus on adult immunization receipt and the systems that support adult immunization. Although mid-adults are at less risk for many infectious diseases as compared to children or older adults, there is still a substantial burden of disease among this population [18, 19]. Furthermore, vaccination is not limited to one phase of life – there is a need to build strong vaccine confidence and improve vaccine receipt across the lifespan. This applies both to those vaccines currently recommend as well as vaccines under development – greater vaccine receipt in young and mid-adults could benefit the introduction of new vaccines for this population as well as receipt of those vaccines already routinely recommended for older adults. Beyond the individual-level factors influencing receipt explored in this study, further research is needed to understand the systems that may influence low coverage in this population, such as lack of provider engagement or recommendations or weak immunization information infrastructure, and how these factors may interact with broader social determinants of health. There needs to be a greater effort to promote vaccination and engagement with the healthcare system among young and mid-adult women.

## Data Availability

The datasets analyzed during the current study are not publicly available give that the parent case-control study is ongoing. However, data will be made available by corresponding author among on reasonable request.
